# Early initiation of complementary feeding practice and its associated factors among children aged 6 to 24 months in Northeast Ethiopia

**DOI:** 10.1186/s41043-024-00554-y

**Published:** 2024-05-16

**Authors:** Yitbarek Wasihun, Getahun Addissie, Muluken Yigezu, Natnael Kebede

**Affiliations:** 1 Department of Health Promotion, School of Public Health, College of Medicine and Health Sciences, Wollo University, Dessie, Ethiopia; 2 Department of Public Health, College of Zemen Postgraduate, Dessie, Ethiopia; 3 Department of Public Health College of Medicine & Health Science, Dire Dawa University, Dire Dawa, Ethiopia

**Keywords:** Early initiation complementary feeding, Determinants, Ethiopia

## Abstract

**Background:**

Even if there is currently no research on food integration practices, there is an unreliable hole in the data for the first motives, especially 6 to 24 months, research at ages varies in different ways, always, but the child’s development affects an important part of both the child and the parents. This gap limits our comprehensive knowledge of strategic choices and—their potential impact on children’s overall health and well-being. Therefore, the aim of this study was factors shaping complementary feeding for 6 to 24-Month-Olds in Northeast Ethiopia.

**Methods:**

A community-based survey was conducted in northeastern Ethiopia from June to July 2022. A sample of 409 mothers with infants aged 6 to 24 months was selected using a simple random sampling method. A structured questionnaire was adopted and data were collected by an interviewer. Collected data were entered into Epi Data version 4.6 and subsequently converted to SPSS version 21 for further analysis. Variables with a *P*-value <  = 0.25 in the bivariate analysis were included in the multivariable logistic regression model. Multivariable logistic regression analyses aimed at identifying independent associations between early initiation of supplement feeding and determinants-adjusted odds ratios with corresponding 95% confidence intervals were calculated to determine the strength of associations. *P*-values less than or equal to 0.05 were considered statistically significant.

**Results:**

A total of 409 mothers with their children were included in the analysis, revealing a prevalence of 38.1% for early initiation of complementary feeding among children aged 6–24 months. Factors associated with -early initiation included place of residence (Adjusted Odds Ratio (AOR) 3.63, 95% Confidence Interval (CI) 1.1–11.95), husband’s educational status (AOR 16.83, 95% CI 1.98–24.8), maternal occupation (AOR 21.2, 95% CI 1.11–46.9), number of antenatal care (ANC) visits (AOR 25.94, 95% CI 22.7–85.67), initial breastfeeding time (AOR 4.98, 95% CI 1.22–14.9), and medical illness (AOR 2.81, 95% CI 1.12–3.6.

**Conclusion:**

Significant associations with Complementary Feeding were identified with the number of antenatal care (ANC) visits, postnatal care (PNC) check-ups, current residency, breastfeeding initiation time, maternal medical illness, and occupational status. To mitigate the early initiation of complementary feeding, it is recommended to enhance ANC/PNC services and educate mothers about the precise timing for introducing complementary foods to their infants.

## Introduction

Early initiation of complementary feeding practice refers to the timely introduction of solid foods and liquids other than breast milk [1]. Globally, only 36% of children are exclusively breastfed from birth to five months of age, while developed countries show higher rates of early introduction of solid foods, ranging from 40.4 to 83.5% [2, 3]. The World Health Organization (WHO) advises exclusive breastfeeding for the first six months of a child’s life. Despite this recommendation, approximately 50% of children aged 4–5 months and around 15% of infants aged 2–3 months are already consuming solid foods in the regions of Latin America and the Caribbean, as well as East Asia and the Pacific [4].

In a birth cohort study conducted in the Netherlands, findings revealed that children introduced to complementary feeding before the age of 4 months had a 1.32 times higher risk of experiencing overweight throughout their childhood compared to those with later initiation of complementary feeding [5]. Additionally, the premature introduction of liquids and solid foods significantly elevates the risk of diarrheal diseases—a leading cause of morbidity and mortality among infants and young children in Africa [6]. In 2018, close to 200 million children under the age of 5 experienced stunting or wasting, with an additional 340 million grappling with hidden hunger [4].

 Malnutrition, whether through direct or indirect means, has been a contributing factor in 60% of the annual 10.9 million deaths among children under the age of five [3, 7]. Inadequate breastfeeding and complementary feeding practices are the primary drivers of undernutrition. Promoting exclusive breastfeeding for the first 6 months of life, followed by continued breastfeeding alongside appropriate complementary feeding, could prevent 13% and 6% of under-5 deaths each year, respectively[3].

In Ethiopia, exclusive breastfeeding is practiced by only 59% of infants below six months. Despite the World Health Organization’s (WHO) recommendation for exclusive breastfeeding in this age group, 14% of infants aged 0–5 months consume plain water, 1% intake non-milk liquids, 8% have other milk, and 13% incorporate complementary foods alongside breast milk. Notably, 6% of infants under six months are not breastfed at all. Despite government efforts, approximately 41% of children under six months of age are introduced early to complementary feeding [8].

Several factors have been identified as potential barriers to early initiation of complementary feeding practices, including socio-demographic factors such as religious ethnicity, age of mothers and infants, family size, maternal age, educational status, maternal occupational status, marital status, and age of the child. Knowledge and practice-related factors such as delayed breastfeeding initiation assistance and inability to know the exact time to start CF also contribute to delayed initiation of complementary feeding practices [9–11].

The study on early initiation of complementary feeding practice and its associated factors among children aged 6 to 24 months in Northeast Ethiopia fills a significant gap by focusing specifically on the region of Northeast Ethiopia. This geographical specificity allows for a nuanced understanding of local factors influencing complementary feeding practices, which may differ from findings in other regions of the country. By identifying these unique factors, the study offers tailored recommendations for interventions aimed at improving early nutrition practices and child health outcomes in Northeast Ethiopia, thereby contributing to the advancement of public health initiatives in the region.

## Methods and materials

### Study area, period, and study design

A Community-based cross-sectional study was undertaken from June to July in northeast Ethiopia.

### Population

All mothers having children 6 to 24 months old in northeast Ethiopia were the source population. All mothers having children 6 to 24 months old in northeast Ethiopia during the data collection period were the study population. All mothers having children with 6 to 24 months old were included in the study. Critically ill individuals unable to communicate during data collection were excluded.

### Sample size, sampling procedures, and techniques

To determine the sample size, various predictors significantly associated with the outcome variable were taken into consideration. Using the single population proportion formula, the sample size was calculated. The total number of infants aged 6–24 months in the six selected clusters in northeast Ethiopia was 782. As this figure falls below 10,000, the following adjustment formula for the sample size was applied. Therefore, n = 409/ (1 + 409/782) n ~ 52.Taking a 10% [12] non-response rate the final sample size was 409. Eight kebeles were randomly chosen from a total of thirty-six in this study conducted in northeast Ethiopia. The sample size for each kebele was proportionally allocated based on the number of children aged 6–24 months in the selected kebeles. Subsequently, a systematic sampling technique was employed using the sampling interval formula (K = N/n), where “N” represents the total number of samples in each kebele, and “n” is the desired sample size. This involved selecting study participants from each kebele by visiting every Kth household. The initial caregiver was chosen through a lottery method.

### Operational definitions and standard definitions

*Early initiation of complementary feeding*; if a baby had received any kind of food or fluids in addition to breast milk before six months of infancy, and categorized as “yes.” Conversely, if a baby had not received any kind of food or fluids in addition to breast milk before six months of infancy, it was categorized as “no.” [13].

*Complementary feeding*; is giving of children’s other foods, fluids, or semifluid in addition to the breast milk [14, 15].

*Complementary feeding timing*; is the introduction of additional supplementary food for young children at six months of age [16], 17.

*Delayed breastfeeding initiation*: If breastfeeding was not initiated immediately after birth (within an hour) it is considered as late breast-feeding initiation [14].

To determine the level of mothers’ knowledge regarding early initiation of complementary feeding, the mean was used as a cut point.

*Good Knowledge*: In assessing knowledge of early initiation of complementary feeding practices among children aged 6 to 24 months in Northeast Ethiopia, seven key items were utilized. Respondents demonstrating a score above the mean across these items were categorized as possessing good knowledge.

*Poor Knowledge*: Respondents would be categorized as having poor knowledge of early initiation of complementary when the respondents scored below the mean knowledge score questions.

*Post-natal care*: The care given to the mother and her newborn baby immediately after birth and for the first six weeks.

*Post-natal period*: Begins immediately after childbirth and extends up to six weeks [18].

*Antenatal care*: is a maternal healthcare service provided by skilled healthcare professionals to pregnant women.

*Early initiate CF*: the time at which the mother starts giving the child either solid, semisolid, or liquids other than breast milk or formula feeding.

### Data collection tools and procedure

Interviews were conducted using semi-structured questionnaires developed in English. The data collection process involved the use of semi-structured, pre-tested, interviewer-administered questionnaires. These questionnaires, adapted from a previous source, comprised seventy items organized into seven domains. The collection of socio-demographic characteristics included eight items, encompassing age, sex, marital status, religion, monthly income, educational status, ethnicity, and occupation.

### Data quality assurance

Data quality was assessed during the design of the questionnaire (data extraction format), data collection, and data entry. A questionnaire (data extraction format) was prepared with the study’s objectives in mind, logically sequenced, and devoid of scientific and technical terms. The data collection format underwent pre-testing on approximately 31 (5%) of study subjects in the nearby kebele. The data collectors and supervisors were trained for three days on the study’s objectives and data quality to reduce inter-individual variability. The collected data was checked daily by the researcher and assigned supervisor for any incompleteness and/or inconsistency. If any incompleteness and/or inconsistency appeared, corrections were made by referring to the registration log book or by taking the appropriate measures.

### Data processing and analysis

Data underwent entry, categorization, coding, and summarization using Epidata version 4.6, and subsequent transformation to SPSS version 21 on the computer-facilitated further analysis. The analysis unfolded in three stages: initially, a descriptive analysis was executed to establish the frequency, percentage, means, and standard deviations of both dependent and independent variables. Following this, bivariate logistic regression was conducted to explore the association between explanatory and outcome variables. Finally, variables with a *P*-value of <  = 0.25 in the bivariate analysis were integrated into a multiple logistic regression model to identify independently significant predictor variables. Adjusted Odds Ratio (AOR) with a 95% confidence interval (CI) was utilized for this determination, and a *P*-value less than or equal to 0.05 was deemed statistically significant.

## Results

### Socio-demographic characteristics of the respondents

In this study, 409 mothers and children were included with a mean age of 29.32 (± 6.1 SD) years and 13.16(± 4.9 SD) months, respectively. Around (57%) of respondents were in the age group of 20–30 years about (37.2%) were in the age group of 31–40 years and 358 (87.5%) were orthodox Christian followers. majority of the women interviewed were married 347(84.8%) and rural dwellers 315(77%). The majority of Women and their husbands were unable to read and write 136(33.3%) and 139(34%) respectively. About 174(42.5%) of the respondents can read and write, 28 (6.8%) had primary school level and 15(3.7%) had secondary school level, the percentage of Mothers who had college and university level was 56(13.7%). The occupational status of Mothers and her husband had 283(69.2%) house wives and 251(6.4%) farmer and child sex is male and female 247(60.4%) and 162(39.6%) respectively (Table 1).

**Table 1:** Socio-demographic characteristics of Lactating women for 6 to 23 months Period Northeast Ethiopia 2022. (n = 409)

Variable		Frequency	Percent%
Age of mother	< 20 years	12	2.9
	20–30 years	233	57
	31–40 years	152	37.2
	> 40 years	12	2.9
Place of Residence	Rural	315	77
	Urban	94	23
Marital Status	Married	347	84,8
	Divorced	35	8.6
	Single	27	6.6
Religion	Orthodox	358	87.5
	Protestant	7	1.7
	Muslim	43	10.5
	Other	1	0,2
Mother’s educational status	Can’t read and write	136	33.3
	Can read and write	174	42.5
	Primary (grade 1–8)	28	6.8
	Secondary (grade 9–12)	15	3.7
	Above Secondary	56	13.7
Mother’s occupational status	Housewife	283	69.2
	Government employee	52	12.7
	Privet employee	8	2.0
	Daily Labor	8	2.0
	Merchant	58	14.2
Husband’s educational status	Can’t read and write	139	34
	Can read and write	159	38.9
	Primary (grade 1–8)	18	4.4
	Secondary (grade 9–12)	28	6.8
	Above Secondary	65	15.9
Husband’s occupational status	Farmer	251	61.4
	Government employee	9	2.2
	Privet employee	11	2.7
	Daily Labor	80	19.6
	Merchant	54	13.2
	Other	4	1
Sex of Child	Male	247	60.4
	Female	162	39.6

### Reproductive health and health service utilization of Lactating women’s

Among 409 mothers, 361 (88.3%) had ANC follow-ups during their pregnancy in their respective health centers. However, their ANC follow-up decreases from the first visit to the consecutive visits. Only 104 (25.4%) of them had four or more times of ANC follow-up. The study also assessed the place and mode of delivery of the current child and the study found that the majority of 231 (56.5%) mothers gave birth at home with traditional birth attendants and almost all 373(91.2%)of them delivered the current child with spontaneous vaginal delivery (SVD). At the time of delivery, 313 (76.5%) mothers received postal natal (PNC) service, and 347 (84.8%)of them took health education about exclusive breastfeeding till 6 months and initiation of complementary feeling after 6 months of child age (Table 2).

**Table 2:** Reproductive health and health service utilization of Lactating women for a 6 to 23-month period Northeast Ethiopia health centers, 2022. (n = 409)

Variable		Frequency	Percent
ANC Visits	Yes	361	88.3
	No	48	11.7
Times of ANC Visits	One time	171	41.8
	Two times	57	13.9
	Three times	75	18.3
	Four times	82	20
	Five and more	24	5.9
Place of delivery for current child	Health institutions	178	43.5
	Home	231	56.5
Mode of delivery	SVD	373	91.2
	Cesarean section	22	5.4
	Instrumental Delivery	14	3.4
Birth preparedness	Yes	318	77.8
	No	91	22.2
Baby weight measure	Yes	310	75.8
	No	99	24.2
PNC service	Yes	313	76.5
	No	96	23.5
No of the children	1–3	349	85.3
	4–6	58	14.2
	7–8	2	0.5
Family size	< 3 members	8	1.9
	3–4 members	241	58.9
	5–6 members	147	36
	≥ 7members	13	3.2
Mothers counseled about the CF during ANC/PNC	Yes	347	84.8
	No	62	15.2

### Mother’s knowledge about complementary feeding

Among 409 women, 347 (84.8%)mothers heard about exclusive breastfeeding (EBF)of which 280 (68.5%) of them knew the exact time of EBF till 6 months of child age. In addition, 365 (89.2%) of mothers heard about the exact time to start complementary feeding and 246 (60.1%) of them got appropriate information about complementary feeding at the health institution level through health education by health professionals. 369 (90.2%) of study participants were breastfeeding their child at the time of interviewing and only 94 (23.0%) out of the total 409 participants perceived to continue breastfeeding until 2 years of child age. And of 409 women, 351 (85.8%) of mothers started complementary feeding at the time of data collection of which 156 (38.1%) of mothers started before 6 months of child age because 117(75.0%) of them perceived their breast milk was not sufficient for their child (Table 3).

**Table 3:** Mothers’ knowledge about complementary feeding Northeast Ethiopia health centers, 2022. (n = 409)

Variable		Frequency	Percent
Mothers heard about complementary feeding	Yes	353	86.3
	No	56	13.7
Mothers heard about the exact time to start the CF	Yes	365	89.2
	No	44	10.8
Sources of information	Health institution	246	60.1
	Health extension workers	131	32
	Radio	5	1.2
	Television	15	3.7
	Others	10	2.4
Feeding anything from a bottle	Yes	248	60.6
	No	161	39.4
Mothers hearing about exclusive breastfeeding	Yes	347	84.8
	No	62	15.2
The recommended duration of exclusive breastfeeding	Six months or less	280	68.5
	6–11 months	42	10.3
	12–23 months	47	11.5
	≥ 24 months	39	9.5
	Don’t know	1	0.2
The recommended time to stop breastfeeding	Up to 6 months	226	55.3
	6–11 month	55	13.4
	12–24 month	94	23
	Don’t now	34	8.3
Currently breastfeeding status	Yes	369	90.2
	No	40	9.8
Initial breast-feeding time	Within 1 h	322	78.7
	After an hour	80	19.6
	After a day	7	1.7
Complimentary feeding	Yes	351	85.8
	No	58	14.2
Initiation time of complementary feeding	< 6 months	156	38.1
	≥ 6 months	253	61.9
Reason for starting CF < 6 months of age	My breast milk is not sufficient	117	75.0
	Due to maternal medical illness	15	9.6
	Lack of information	15	9.6
	My workplace is far from my house	2	1.3
		7	4.5

### Factors associated with early initiation of complementary feeding

Bivariate analysis showed that maternal and husband educational status, maternal occupation, age of mother, number of ANC visits, Place of delivery, mode of delivery, PNC service, counseling on appropriate CF practices during ANC/delivery//PNC visit, mass media exposure, Feeding anything from a bottle, Heard about exclusive breastfeeding, How long advice breastfeeds duration, knowing the recommended time of CF initiation, HIV test, medical and breast illness were Candidate variables for multiple binary logistic regression analysis at a *p*-value of <  = 0.25. However, the multiple binary logistic regression analysis identified, that only six variables (place of current residence, numbers of ANC visits, medical illness, maternal occupation, Initial breastfeeding time, and husband education) were significantly associated with the independent variable. The study showed that those mothers from urban residences were 3.63 times more likely to early initiated CF compared to those from Rural residences [AOR: 95% CI 3.63 ((1.1–11.95)], Housewife mothers were 21.2 times more likely to introduce complementary foods early to their infants than Government employed mothers [AOR (95%CI) = 21.2 (1.11, 46.9] (Table 4).

**Table 4:** Factors associated with early initiation of complementary feeding among infants aged 6–23 months in northeast Ethiopia, 2022 (n = 409)

Variable	Category	Early initiation of CF		COR ( 95% CI)	*P*-value	AOR ( 95% CI)
		Yes	No			
Age [mothers]	<30	99	146	1	0.48	1
	> 30	57	107	0.78 (0.521–1.18)	0.06	1.15(0.61–2.16)
Place of residence	Urban	66	28	5.89(3.55–9.76)	0.03	3.63(1.1–11.95)*
	Rural	90	225	1	0.0	1
Maternal educational status	Unable to read and write	44	92	5.22 (2.64–10.33)	0.56	0.45(0.03–6.39)
	Able to read and write	49	125	6.37 (3.27–12.43)	0.99	1.01(0.08–12.02)
	Primary school	11	17	3.86 (1.49–10.03)	0.84	1.34(0.08–22.05)
	Secondary school	12	3	0.63 (0.15–2.51)	0.29	4.73(0.25–87.72)
	Diploma and above	40	16	1	0.32	1
Husband educational status	Unable to read and write	36	103	8.76 (4.44–17.29)	0.01	16.83(1.98–24.8)*
	Able to read and write	43	116	8.26 (4.25–16.05)	0.001	38.57(4.83–55.25)*
	Primary school	7	11	4.81 (1.59–14.5)	0.02	23.2(1.75–36.44)*
	Secondary school	21	7	1.02 (0.36–2.85)	0.99	0.99(0.19–37.73)
	Diploma and above	49	16	1	0.01	1
Occupation of mothers	Housewife	84	199	16.58(2.01–136.9)	0.04	21.2(1.11–46.9)*
	Merchant	24	34	9.92(1.14–85.94)	0.06	18.06(0.86–38.74)
	Private employee	7	1	1.0(0.05–19.36)	0.03	12.85(1.45–13.8)*
	Daily labor	7	1	3.71(0.42–32.52)	0.03	5.46(7.19–16.84)*
	Government employee	34	18	1	0.04	1
Number of ANC visits,	1visit	15	156	18.4 (27.42–39.2)	0.0	25.94(22.7–85.67)*
	2 visit	33	24	15.27(1.92–11.5)	0.011	39.08(2.33–44.66)*
	3 visit	42	33	16.5(2.1–12.1)	0.005	5.66(3.17–8.4)*
	4 visit	44	38	8.13(2.33–11.23)	0.023	22.71(1.53–33.03)*
	5 and more visit	21	2	1	0.0	1
Place of delivery	Health institution	46	132	2.61(1.71–3.98)	0.7	0.83(0.33–2.09)
	Home	110	121	1	0.47	1
Mode of delivery	SVD	135	238	3.17(1.04–9.66)	0.08	0.14(0.02–1.29)
	CS	12	10	1.5(0.38–5.95)	0.06	0.09(0.01–1.13)
	Instrumental	9	5	1	0.16	1
PNC service	Yes	110	203	0.59(0.37–0.94)	0.56	0.68(0.21–2.25)
	No	46	50	1	0.00	1
Counseling on appropriate CF practices during ANC /PNC visit	Yes	127	220	1.52 (0.88–2.62)	0.56	1.53(0.37–6.24)
	No	29	33	1	0.62	1
Sources of information	Health institution	78	168	5.03(1.26–19.95)	0.21	4.68(0.42–52.57)
	Health extension workers	52	79	3.55(0.87–14.33)	0.28	3.61(0.34–38.42)
	Radio	5	0	0.0(0)	0.99	0.0
	Television	13	2	0.36(0.05–2.68)	0.83	35.81(0.06–1.44)
	Others	7	3	1	0.62	1
Feeding anything from a bottle	Yes	76	172	2.24(1.48–3.37)	0.23	1.83(0.69–4.85)
	No	80	81	1	0.94	1
Mothers hearing about exclusive breastfeeding	Yes	121	226	2.42(1.39–4.19)	0.002	6.88(1.39–11.65)*
	No	35	27	1	0.31	1
The recommended time to stop breastfeeding	Up to 6 months	72	154	2.4(1.16–4.98)	0.038	0.22(0.04–1.15)
	6–11 month	27	28	1.16(0.49–2.74)	0.089	0.59(0.09–3.71
	12–24 month	39	55	1.58(0.72–3.49)	0.09	1.39(0.09–1.72)
	Don’t now	18	16	1		1
Initial breast-feeding time	Within 1 h	102	220	12.94(1.53–18.8)	0.038	4.98(1.22–14.9)*
	After an hour	48	32	4(0.46–34.8)	0.089	5.76(0.64–18.63)
	After a day	6	1	1	0.09	1
Medical illness	Yes	74	60	0.34(0.23–0.53)	0.003	2.81(1.12–3.65)*
	No	82	193	1	0.0	1
Tested for HIV	Yes	144	249	0(0)	0.24	2.81(0.51–15.58)
	No	12	4	1	0.00	1

*significant at* p* value less 0.05

## Discussion

This study identified five factors (place of current residence, numbers of ANC visits, medical illness, maternal occupation, and husband education) that were associated with Early Initiation of complementary feeding. The maternal occupation had a significant role in the Early Initiation of complementary feeding in the first 6 months. Housewife mothers were more likely to introduce complementary foods early to their infants than government-employed mothers in Ethiopia. Similarly, a study conducted in Ghana found that maternal education, occupation, and place of residence were associated with early initiation of complementary feeding [19]. In contrast, a study conducted in India found that maternal education and occupation were not significant predictors of early initiation of complementary feeding, but instead, factors such as birth order and type of delivery were associated with early initiation [20]. The possible discrepancy reason for the difference in findings between the Ethiopian and Indian studies could be due to differences in cultural and societal norms surrounding infant feeding practices. In Ethiopia, it may be more common for housewife mothers to introduce complementary foods early due to cultural beliefs or economic factors, while in India, other factors such as birth order and type of delivery may play a more significant role. Additionally, differences in study design, sample size, and data collection methods could also contribute to the differing results.

The findings of this study also indicated that mothers who did not attend PNC had a 68% increased risk of initiating complementary foods early when compared to mothers who attended PNC [AOR (95% CI) = 0.68 (0.21–2.25)]. A study conducted in rural Gujarat, India found that mothers who did not attend PNC were more likely to introduce complementary foods early [21]. Overall, the findings suggest that PNC attendance may be an important factor in promoting optimal infant feeding practices.

Mothers who received counseling on complementary feeding during their antenatal care visits were more likely to initiate complementary feeding at the appropriate time compared to those who did not receive such counseling. Additionally, mothers who attended antenatal care services were more likely to initiate complementary feeding practices than those who did not attend. This result is similar to the findings in India [22]. One possible justification for these findings is that counseling during antenatal care visits provides mothers with important information and education about the benefits and timing of complementary feeding. This knowledge may increase their confidence and motivation to initiate complementary feeding at the appropriate time. Additionally, attending antenatal care services may indicate a greater level of engagement with healthcare providers and a greater awareness of the importance of early childhood nutrition, which may also contribute to higher rates of complementary feeding initiation. The similarity of these findings with those from India suggests that this relationship between counseling, antenatal care attendance, and complementary feeding initiation may be consistent across different cultural contexts.

## Conclusion and recommendations

In summary, Significant associations with Complementary Feeding were identified with the number of antenatal care (ANC) visits, postnatal care (PNC) check-ups, current residency, breastfeeding initiation time, maternal medical illness, and occupational status. To mitigate the early initiation of complementary feeding, it is recommended to enhance ANC/PNC services and educate mothers about the precise timing for introducing complementary foods to their infants. In addition, our quantitative study on early initiation of complementary feeding highlights valuable insights. We suggest future research blend quantitative data with qualitative exploration to delve into caregivers’ experiences and reasons, enriching understanding and informing targeted interventions for optimal child nutrition and health outcomes.

## Data Availability

All the necessary data are included in the manuscript. An English version data collection tool and detailed operational definitions of the outcome variable are accessible at a reasonable request from the corresponding author**.**

## Abbreviations

ANC: Antenatal care
AOR: Adjusted odd ratio
BF: Breast feeding
BSC: Bachelor of science
C/S: Cesarean section
COR: Crude odd ratio
C.F: Complementary feeding
DHS: Demographic and health survey
EBE: Exclusive breast feeding
MPH: Master of public health
SNNPR: South nation nationality of people of representative
PNC: Post-natal care
WHO: World health organization
RH: Reproductive health
Ph.D.: Public health doctor
SPSS: Statically package social science
WFP: World food program
IRB: Institutional review board
SAM: Sever acute malnutrition
MAM: Moderate actuate malnutrition
OTP: Outpatient program
SC: Stabilization center
